# Prediction of scaffold proteins based on protein interaction and domain architectures

**DOI:** 10.1186/s12859-016-1079-5

**Published:** 2016-07-28

**Authors:** Kimin Oh, Gwan-Su Yi

**Affiliations:** Department of Bio and Brain Engineering, KAIST, Daejeon, Korea

## Abstract

**Background:**

Scaffold proteins are known for being crucial regulators of various cellular functions by assembling multiple proteins involved in signaling and metabolic pathways. Identification of scaffold proteins and the study of their molecular mechanisms can open a new aspect of cellular systemic regulation and the results can be applied in the field of medicine and engineering. Despite being highlighted as the regulatory roles of dozens of scaffold proteins, there was only one known computational approach carried out so far to find scaffold proteins from interactomes. However, there were limitations in finding diverse types of scaffold proteins because their criteria were restricted to the classical scaffold proteins. In this paper, we will suggest a systematic approach to predict massive scaffold proteins from interactomes and to characterize the roles of scaffold proteins comprehensively.

**Results:**

From a total of 10,419 basic scaffold protein candidates in protein interactomes, we classified them into three classes according to the structural evidences for scaffolding, such as domain architectures, domain interactions and protein complexes. Finally, we could define 2716 highly reliable scaffold protein candidates and their characterized functional features. To assess the accuracy of our prediction, the gold standard positive and negative data sets were constructed. We prepared 158 gold standard positive data and 844 gold standard negative data based on the functional information from Gene Ontology consortium. The precision, sensitivity and specificity of our testing was 80.3, 51.0, and 98.5 % respectively. Through the function enrichment analysis of highly reliable scaffold proteins, we could confirm the significantly enriched functions that are related to scaffold protein binding. We also identified functional association between scaffold proteins and their recruited proteins. Furthermore, we checked that the disease association of scaffold proteins is higher than kinases.

**Conclusions:**

In conclusion, we could predict larger volume of scaffold proteins and analyzed their functional characteristics. Deeper understandings about the roles of scaffold proteins from this study will provide a higher opportunity to find therapeutic or engineering applications of scaffold proteins using their functional characteristics.

**Electronic supplementary material:**

The online version of this article (doi:10.1186/s12859-016-1079-5) contains supplementary material, which is available to authorized users.

## Background

Cells regulate and integrate various functional modules to monitor external and internal states, and to execute the appropriate physiological responses. Generally, cells can monitor environmental stimuli using sensors like receptors. This information is then processed by intracellular signaling networks to control various cellular outputs. Scaffold proteins are known as an important controller in this process [1]. Scaffold proteins are signaling organizers which can modulate signaling specificity, integration, crosstalk, feedback, and multiplicity by acting as a physical platform to assemble signaling components [2, 3]. Through these regulations, scaffold protein can lead to dynamic signaling outputs [4]. Scaffold proteins are involved not only signaling processes but also in the assembly-line processes and cell-cell communications [1]. Scaffold proteins also control enzymatic activities by conformational fine-tuning. Scaffold proteins can engage their interacting partners and transport them into specific cellular compartments [5]. To sum up, scaffold proteins basically need to assemble multiple proteins by protein-protein interaction using interacting domain to enforce proximity. Mainly, scaffold proteins regulate spatial organization of reactions and control dynamics by recruiting modifiers or acting as catalysts. They also act as a signaling/metabolism organizer. Through these functionalities of scaffold proteins, it is possible to combine the use of these elements, protect activated signaling molecules from inactivation, and control dynamic signaling output.

As mentioned, the characteristics of scaffold proteins could be applied as therapeutic targets to treat human diseases and industrial applications to synthesize desired chemical products by engineering. There has been encouraging example of scaffold proteins as therapeutic applications. Some studies have suggested IQGAP1 proteins are highly expressed in cancer cell lines [6] and plays a role for scaffold protein IQGAP1 in enhancing tumorigenesis, but IQGAP1 knockout mice are viable and fertile, do not show any defects in normal epithelium and heal wounds normally [7]. Thus, IQGAP1 is a potential tumor-required scaffold protein that is dispensable for homeostasis. So, they made scaffold-kinase interaction blockade (SKIB). SKIB acts using a mechanism distinct from direct kinase inhibition and may be a strategy to target overactive oncogenic kinase cascades in cancer [8]. Like this example, aberrant regulation of these various cellular functions can lead to the development of many types of diseases, because scaffold proteins act as systemic regulators in cellular network.

In spite of the importance of scaffold protein, only a few have been discovered on an individual basis and their regulatory roles are largely unknown. Zeke et al. provide a definition for classical scaffold proteins. Classical scaffold protein can be defined as proteins that: (i) lack intrinsic catalytic activity relevant for signaling; (ii) have at least two binding partners with catalytic activity relevant for signaling; and (iii) have binding partners that interact with each other in a direct or indirect way [4]. Fidel and Mario firstly predicted potential scaffold proteins from interactomes according to the criteria by Zeke et al. [9]. However, there was a limitation to find diverse scaffold proteins because their criteria were restricted to the classical scaffold proteins. In this study, we searched known scaffold proteins from articles and database and used that knowledge to give reliability to scaffold proteins predicted from interactomes.

We newly defined criteria for finding scaffold proteins focused on structural features to act as scaffold proteins. We extracted 10,127 proteins which have multiple interacting partners from protein interactomes and defined 2716 reliable scaffold proteins according to our novel criteria. We carried out the functional association between scaffold proteins and their recruited proteins and the disease association were tested. Through functional enrichment analysis, we could identify the information of their known function and additional novel implications. As a result, our discovery can help further investigation to study or utilize scaffold proteins for engineering and therapeutics.

## Methods

### Data collection

#### Collection of interactome data

To predict scaffold proteins from interactomes by using structural features, we collected protein-protein interaction (PPI), domain-domain interaction (DDI), and protein-domain, and protein complex data. The protein domain information were taken from the Pfam database [10]. PPI and DDI data were collected from integrated PPI database [11] and integrated DDI database (IDDI) [12] respectively. Moreover, we downloaded the protein complex datasets from COFECO [13].

#### Collection of functional categories

From the UniProtKB, we first obtained totalhuman proteins in SwissProt [14]. Disease-associated genes were collected from three databases: OMIM, PharmGKB [15], KEGG DISEASE [16]. Because the naming of disease status vary among the source databases, we standardized the disease names by extracting the Unified Medical Language System (UMLS) [17] IDs using MetaMap. The UMLS IDs were converted to ICD-10-CM (International Classification of Diseases, 10th Revision, Clinical Modification) once more, using the mapped information that were provided in the UMLS. Drugs and their targets data were collected from DrugBank [18]. To compare the functional associations between scaffolds and their partner proteins, we prepared data of localization and pathway. Localization data is collected from Gene Ontology [19] Cellular Compartment. Each identifier of the Gene Ontology Cellular Compartment is re-organized into 17 cellular compartments according to the hierarchical structure (cell surface, chromosome, cytoplasm, cytoplasmic membrane-bounded vesicle, cytoskeleton, cytosol, endoplasmic reticulum, endosome, ER-Golgi intermediate compartment, extracellular region, Golgi apparatus, mitochondrion, nucleus, plasma membrane, ribosome, sarcoplasmic reticulum, vacuole). Pathway data is collected from four different pathway databases (KEGG [20], PID [21], Reactome [22], and WikiPathway [23]) and defined pathway names and their components were extracted.

#### Collection of known scaffold proteins

To test reliability of our prediction, we defined the gold standard positive and the negative set using basic text mining and functional term filtering. For the gold standard positive set, we collected scaffold protein candidates from multiple sources. First, we manually gathered scaffold proteins from review articles. Second, we found candidates using query search from functional descriptions of UniProt database and title/abstract of PubMed. From those candidates, we filtered out candidates which already have their known molecular functions as scaffold activities and complex assemblies. For the gold standard negative set, we excluded proteins which have molecular functions and biological functions related to known scaffold proteins.

### Criteria for predicting novel scaffold proteins

**Fig. 1:**
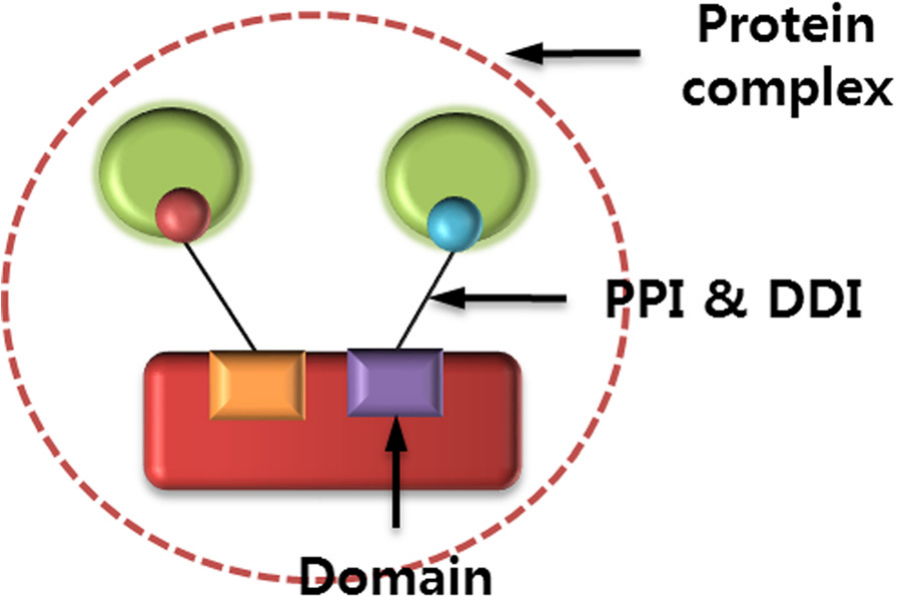
Structural criteria for predicting scaffold protein candidates

We proposed criteria for finding scaffold protein candidates: (i) direct interaction with at least two proteins, (ii) domain-domain interaction between scaffold and two partner proteins using different domain regions, and (iii) scaffold and two partner proteins should be components in the same protein complex (Fig. 1). This criteria is different from Zeke’s definition of classical scaffold protein [4]. Our criteria can filter out hub proteins which have multiple competitive interacting partners using same domain regions.

### Characterization of scaffold protein candidates

#### Gene annotation enrichment analysis

We used the DAVID [24] tools to analyze functional characteristics of collected scaffold protein candidates. The functional meaning of the scaffold protein candidates was interpreted using function enrichment analysis tool in DAVID. We analyzed functional implications in GO molecular function, GO cellular compartment, GO biological process and Pfam families. The p-values were adjusted by multiple testing corrections using Benjamini and Hochberg’s method [25].

#### Functional association analysis

We tested the hypothesis of having no association between scaffold proteins and disease related genes (disease genes or drug targets). To use chi-square statistics, we made contingency table. Observed frequency is compared to expected frequency. If there was no association between scaffold proteins and disease related genes, then the expected frequency should be almost equal to the observed frequency, and the value of the chi-square statistic would be small and the probability (p-value) would be large.

## Results

### Statistics

#### Data statistics

We collected various kinds of resources and constructed database using Oracle 10 g. All proteins were filtered in Homo sapiens and Swiss-Prot which are manually annotated and reviewed. Protein-protein interaction data was filtered by experimental detection methods. Domain-domain interactions were selected which have 3D structural evidences (Table 1-a). We predicted scaffold protein candidates and classified into three types, according to the eligibility criteria. Actually our novel criteria means type I case, however we allowed to classify into type II and type III, because our resources of domain, DDI, and protein complexes were not completely detected (Table 1-b). Both criteria 2 and 3 make scaffold proteins possible to be exist with their partner proteins together simultaneously.

**Table 1:** Statistics

a) Statistics of collected data				
Type	Source			Statistics
Protein	UniProt			Protein: 20233 Articles: 269469
Protein-protein interaction	ComBiCom (BIND, BioGRID, DIP, HPRD, IntAct, MIPS)			Proteins: 82894 PPIs: 73743
Domain	Pfam			Domains: 4895 Proteins: 17316
Domain-domain interaction	iDDI (3DID, iPfam)			Domains: 3214 DDIs: 17770
Protein complex	COFECO			Protein complexes: 3317 Proteins: 4597
Pathway	KEGG, NCI-PID, Reactome, WikiPathway			Pathways: 2620 Proteins: 8413
Cellular location	Gene Ontology Cellular Compartment			GO terms: 635 Proteins: 9820
Disease gene	OMIM, PharmGKB, KEGG Disease			Disease: 502 Genes: 4950
Drug target	DrugBank			Drugs: 1574 Proteins: 1077
Gold standard set	UniProt, PubMed, Gene Ontology			Positive: 104 Negative: 844
Kinase	PhosphoELM, Phosphosite			Kinase: 468
b) Statistics of scaffold protein candidates				
Class	Criteria 1	Criteria 2	Criteria 3	# of scaffold proteins
Type I	O	O	O	616
Type II	O	O	X	1792
Type III	O	X	O	308

### Performance test

To evaluate the ability of the prediction performance, we used a statistical measurement. We defined the gold standard positive and negative scaffold protein set and calculated the number of true positive, false positive, true negative and false negative. Using these four outcomes, we made 2 × 2 contingency table and we obtained precision, sensitivity and specificity of our tests (Table 2). The precision, sensitivity and specificity of our tests were 80.3, 51.0, and 98.5 %, respectively.

**Table 2:** 2 × 2 contingency table for evaluating the performance of prediction

		True condition		
	Total population	Condition positive (158)	Condition negative (844)	Prevalence 17.1 %
Predicted condition (2716)	Predicted condition positive	67	13	Precision (Positive predictive value) 83.8 %
	Predicted condition negative	91	831	False omission rate 9.8 %
	Accuracy (89.6 %)	Sensitivity (True positive rate) 42.4 %	Fall-out (False positive rate) 1.5 %	
		Miss rate (False negative rate) 57.6 %	Specificity (True negative rate) 98.5 %	

### Functional characteristics of predicted scaffold proteins

#### Enriched functions

**Fig. 2:**
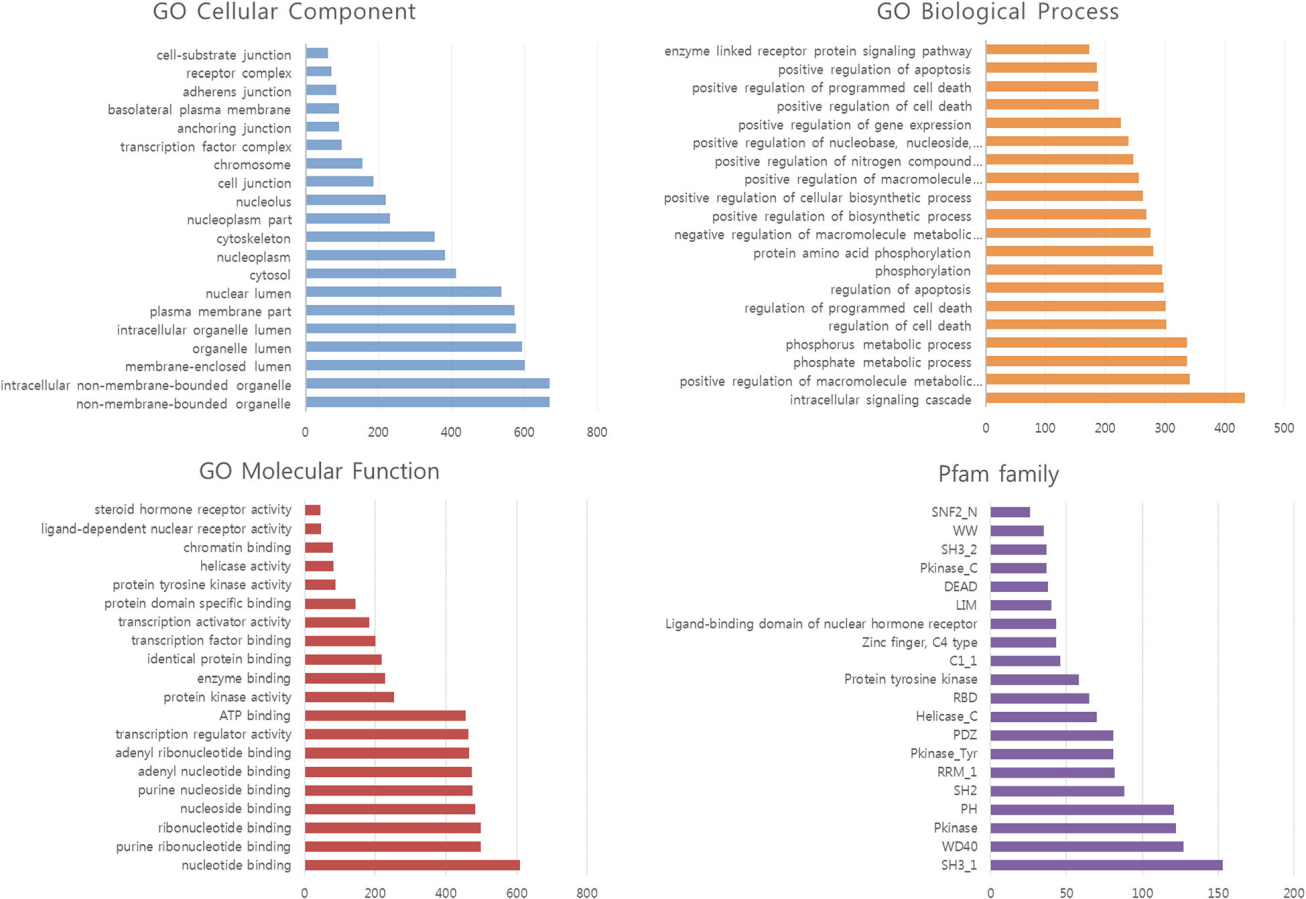
Enrichment of Gene Ontology annotations and Pfam families. The four histograms show significantly enriched Gene Ontology annotations and Pfam domain families for the 2716 scaffold protein candidates. The x-axis represents the number of scaffold protein candidates belonging to the respective category

We carried out a function enrichment analysis for the candidates of scaffold proteins using the GO cellular component (GOCC), GO biological process (GOBP), GO molecular function (GOMF) and Pfam family at Bonferroni corrected p-value of 0.001. Functional enrichment result showed that 87 GOCC, 284 GOBP, 85 GOMF, and 41 Pfam terms are significantly enriched. We could find significant functional implications in the scaffold proteins like ‘metabolic process’, ‘phosphorylation’, ‘cell death’, ‘cell proliferation’, ‘apoptosis’, ‘signaling pathway’, ‘complex assembly’. According to the cellular component result, scaffold proteins had significant enrichments on the all cellular compartments. As we expected, binding that are related to the various molecular functions were significantly enriched. Interestingly, some molecular functions (‘transcription regulator activity’, ‘nucleotide binding’, ‘uniquitin protein ligase binding’) could show that scaffold proteins might have special cellular functions such as assembling transcription factor complex or ubiquitin ligase complex. Furthermore, ‘kinase activity’ shows that the scaffold proteins canalso have catalytic activities and this is distinguished from characteristics of classical scaffold proteins. Well-known modular PPI domains are enriched from Pfam family and it supports binding functions of scaffold proteins (Fig. 2).

#### Functional similarity between scaffold and partner proteins

We compared functional information of scaffold proteins with their partner proteins. In case of Type I, 93.0 % of the total scaffold proteins in Type I have cellular localization information and 99.3 % are matched with partner’s information (Table 3-a). In the same way, 86.2 % of total scaffold proteins in Type I belong to pathways and 96.1 % have partner proteins which have same pathway information (Table 3-b). This result shows the possibilities to predict novel cellular functions of scaffold proteins and partner proteins from their known information.

**Table 3:** Similarity between scaffold protein candidates and partner proteins

a) Similarity of cellular localization			
Type	# of scaffold proteins		
	Total	Known	Matched with partner’s information
Type I	616	573 (93.0)	569 (99.3)
Type II	1798	1372 (76.3)	1285 (93.7)
Type III	308	264 (85.7)	251 (95.1)
b) Similarity of related pathway			
Type	# of scaffold proteins		
	Total	Known	Matched with partner’s information
Type I	616	531 (86.2)	497 (93.6)
Type II	1798	1178 (65.5)	856 (72.7)
Type III	308	212 (68.8)	142 (67.0)

#### Disease association

Some studies have suggested the scaffold protein IQGAP1 as a therapeutic target for inhibiting tumorigenesis. Like this example, scaffold proteins could be disease markers or drug targets because of their important role as a systemic regulator. Hence we tested associations between scaffold proteins and disease related genes. Additionally, we tested associations between set of kinases and disease related genes for comparison. Among 616 scaffold proteins in Type I, 188 scaffold proteins are known as disease genes and 61 scaffold proteins are drug targets. In kinase case, 136 kinases are known as disease genes and 92 kinases are drug targets among total 468 kinases. We made contingency tables about observed and expected frequency. From these contingency tables, we could calculate chi-square values. Table 4 shows that the disease association of scaffold proteins is higher than kinases. Conversely, drug target association of scaffold proteins is lower than kinases, but this result is obvious because kinases have been researched as drug target candidates until now. Our result shows that scaffold proteins have association with diseases and drug targets, so it gives us the reason to study scaffold proteins as therapeutic targets.

**Table 4:** Disease and drug target association of scaffold protein candidates and kinases

	Disease association		Drug target association	
	Scaffold	Kinase	Scaffold	Kinase
Risk ratio	1.26	1.19	1.91	3.94
Odd ratio	1.37	1.27	2.01	4.66
Chi-square value	12.6	5.47	89.12	195.35
*p*-value	3.85E-04	1.93E-02	2.72E-07	2.16E-44

### Case study

**Fig. 3:**
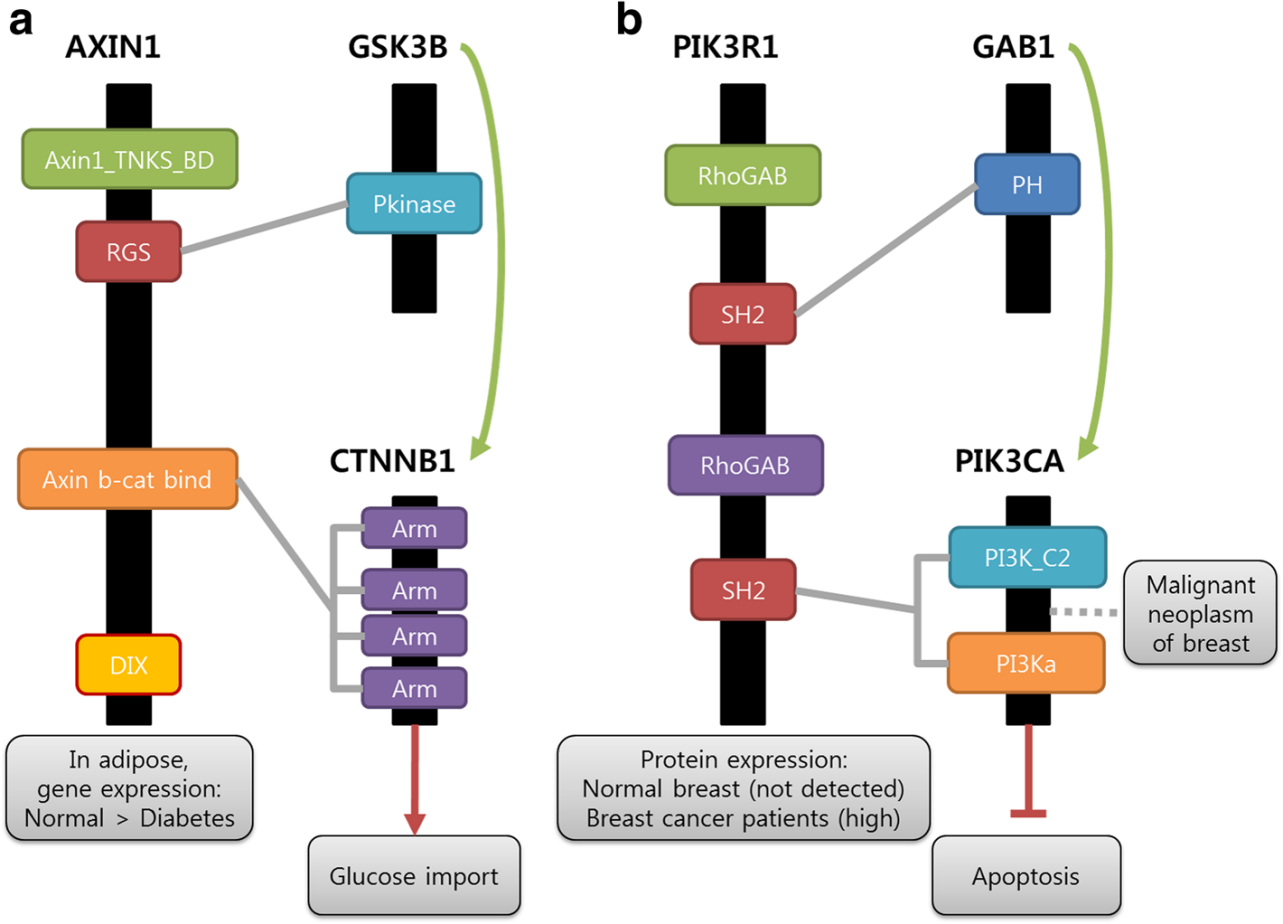
Models of AXIN1 scaffold protein and PIK3R1 scaffold protein candidate. **a** AXIN1 is a known scaffold protein and AXIN1 interacts with GSK3B and CTNNB1 using RGS and Axin b-cat bind domain respectively. CTNNB1 is related activation of glucose import. Through gene expression analysis, AXIN1 is down regulated in type 2 diabetes. **b** PIK3R1 is predicted as a scaffold protein. RIK3R1 can recruit GAB1 and PIK3CA using SH2 domains. PIK3CA is known as a gene related to malignant neoplasm of blast and inhibits apoptotic function. Protein expression of PIK3R1 is not detected in normal breast cell, however it is highly expressed in breast cancer cell

As mentioned, predicted scaffold proteins show high association with disease gene and drug targets. Through the additional analysis, we selected two cases that are related to disease condition from scaffold protein candidates (Fig. 3). AXIN1 is already known as a scaffold protein [26] and in our prediction, it interacts with GSK3B and CTNNB1. We analyzed a microarray dataset in case–control designed from the NCBI Gene Expression Omnibus for type 2 diabetes (GSE29231) [27]. The statistical analysis of gene differential expression was computed and then the p-values of each gene were obtained using the Benjamini & Hochberg method. AXIN1 is down regulated in diabetes condition and CTNNB1 activation is associated with an increment in glucose uptake [28]. From these evidences, we could make hypothesis that type 2 diabetes is caused by a decrement of glucose import because activation of CTNNB1 is inhibited by lower expression of AXIN1.

Our prediction identified PIK3R1 as a scaffold protein candidate by recruiting GAB1 and PIK3CA. We could find protein expression level of PIK3R1 in both the normal cell and the cancer cell using Human Protein Atlas [29]. Protein expression of PIK3R1 was not detected in normal breast cell, however it was highly expressed in breast cancer cell. PIK3CA is known as a gene related malignant neoplasm of breast [30] and inhibits apoptosis function. From these evidences, we could make hypothesis that cancer-specific high expression of PIK3R1 increases activation of PIK3CA and as a result, negatively regulated apoptotic function cause cancer in breast.

## Discussion

Using massive data from high-throughput screening, we could predict plenty of candidate proteins which may act as scaffolds. Many of them are not known as scaffold proteins but they have possibilities to recruitpartner proteins and regulate their functions. Although our text mining methods can be improved, known scaffold proteins extracted from articles and database might be quite helpful to corroborate the reliability of scaffold proteins that are predicted from interactomes. In this study, we used highly reliable data of protein-protein interaction and domain-domain interaction. Because there are many predicted information of protein domain, protein-protein interaction and domaindomain interaction, there is a chance to expand predicted scaffold proteins with scores of reliabilities. If we could utilize functional information or condition specific data, predicted scaffold proteins might be classified into various types by their functional characteristics, such as localization, pathway regulation or crosstalk. These functional characteristics also can be used as a measurements of the reliability scores. Some of known scaffold proteins recruit more than two proteins, but we restricted scaffold protein with two partner proteins, because there are so many possible combinations of partner proteins sets. We can filter and find scaffold proteins which can recruit more than two proteins from our predictions.

## Conclusion

Scaffold proteins can precisely control the specificity and dynamics of information transfer. Furthermore, scaffold proteins have versatility due to their modularity, which allows recombination of protein domains to build new signaling pathways. In the past, scaffold proteins were discovered only by chance via experiments aimed at studying the function of signaling enzymes or receptors. We carried out extraction of scaffold proteins from articles and database and prediction from interactomes according to the new criteria we proposed. Through functional enrichment analysis, we identified not only the known functional implications of scaffold proteins but novel enriched terms. Using functional characteristics of partner proteins, we also predicted new function of scaffold proteins. Finally, we found that scaffold proteins were highly associated with diseases and drug targets like kinases. Through future studies, more can be understood about the role of scaffold proteins, and scaffolds can be used to generate new and predictable pathway to program useful cellular behaviors. In this respect, this study can support further researches for discovering the target of molecular engineering and therapy.

## Ethics approval and consent to participate

Not applicable.

## Consent for publication

Not applicable.

## Availability of data and materials

The datasets supporting the conclusions of this article are included within the article and its additional files (Additional files 1 and 2).

## Additional files

Additional file 1: It contains gold standard positive set, gold negative set and predicted scaffold proteins with type. (XLSX 106 kb) Additional file 2: It contains results of functional enrichment using DAVID. (*P*-value < 0.001). (XLSX 209  kb)
